# Epidemiological Study of Self-Immolation at Khatamolanbia Hospital of Zahedan

**DOI:** 10.5812/ijhrba.13170

**Published:** 2014-03-10

**Authors:** Mostafa Dahmardehei, Fatemeh Behmanesh Poor, Gholamreza Mollashahi, Sedigheh Moallemi

**Affiliations:** 1 Department of Plastic Surgery, Zahedan University of Medical Scinces, Zahedan, IR Iran; 2 Department of Nursing and Midwifery, Zahedan University of Medical Scinces, Zahedan, IR Iran; 3 Deputy of Research, Zahedan University of Medical Sciences, Zahedan, IR Iran

**Keywords:** Self-injury, Epidemiologic Studies, Iran

## Abstract

**Background:**

Self-immolation is a high risk behavior and a way of life termination. Self-immolation, as the most painful of all forms of suicide, is not a common form of suicide in European countries. However, it is highly prevalent in developing countries particularly in Asia and Africa.

**Objectives:**

The aim of this study was to assess the rate and leading factors of self-immolation and gender, geographical distribution, and social norms of affected patients referred to Khatamolanbia Hospital of Zahedan from March 2010 to May 2012.

**Materials and Methods:**

This descriptive and objective-based study was cross-sectional and retrospective with 750 burn patients; 315 of them had attempted self-immolation and most of them died. The data collection tool was a two partite questionnaire consisting of 17 questions (8 questions about personal details and 9 questions about self-immolation factors).

**Results:**

350 self-immolations resulting in death were reported in this study; these subjects were 16-25 years old, 67.25% female, 63.55% elementary school education, 74.20% married, 69.10% housewife, 61.05% resident of Saravan, 93.35% oil burns, 72.30% middle and low social class, 90.20% burn over 68%, 20% psychiatric illness history and being treated with antidepressants, 73.25% verbal and physical violence before burning, and 100% of the burnings took place inside a house and usually during the afternoon.

**Conclusions:**

Due to the high rate of self-immolations in this area, solutions for improvement of life quality and social norms should be reviewed and implemented.

## 1. Background

A review of history of different societies indicates that in most historical periods, people have always wished to preserve their rights to remain alive or dead by some means. Furthermore, it is possible to identify the reasons, which have driven people to commit suicide in a given society and a specific era by considering their cultural and social status and the conditions dominating their lives. In the recent years, people’s awareness and collective perception of suicide have changed to an extent that some regard it as a logical and reasonable way to respond to problems. With the alarming increase in the rate of suicide and its social, economic and psychological consequences, the World Health Organization (WHO) identified “a global decline in suicide rate by 2000” as one of its goals in its general health initiative (1).

Self-immolation is a high risk behavior and a way of life termination. As a social problem, it imposes large losses and costs to the individual, their family and society. In religious teachings, suicide is disobedience of God. Citing to verse 32 of Sura Maida, Imam Sadiq states “whoever intentionally commits suicide will be forever in hell” (2). Self-immolation is a deadly, destructive, painful and costly issue for the individual and the society. Those who set themselves on fire are hardly treated and consequently suffer serious physical and spiritual complications. Limitation of motion and skin lesions, among other things adds insult to injury. Self-immolation is among the most appalling violent affairs, many dimensions of which are still unknown. According to statistics, globally about 500,000 people lose their lives due to suicide annually. Suicide basis is 45,000 individuals in Sweden and 58,000 individuals in Hungary per 100,000 people. In Iran suicide rate is 5-18.22%. Self-immolation, as the most painful of all forms of suicides, is not a common form of suicide in European countries (3). However, it is highly prevalent in developing countries particularly in Asia and Africa. Some studies indicate a high rate of self-immolation in Iran (besides India) with 25-40% of suicides devoted to self-immolation (4, 5). Studies have been performed in some cities of Iran such as Ilam, Kermanshah, Khuzestan, Tehran, and Birjand (3, 5, 6).

A statistical evaluation of self-immolation during 2002 studied 55 countries in 20 years and revealed 3,351 cases of immolation, 2,296 of which died. India has the highest self-immolation rate leading to death, Sri Lanka has the highest self-immolation rate, and United States and European countries have the lowest rates. Men in Western countries and women in the Middle East and India are more likely to attempt self-immolation. Patients in Europe are 10 years younger than Asian patients (7). Analysis suggested that, by 2001, male suicide rate in Iran was lower than the total male suicide cases reported from all countries located in Europe, North America, Oceania and East Asia, and less than cases reported from most countries located in Latin America, Central America and South America; however, the rate of suicide was higher than that of most countries in Western Asia. Yet, comparison with female suicide rate in other countries revealed a relatively different trend for Iran. The rate was higher than that of all countries in eastern, western and northern Europe, North America and Oceania and most countries located in southern Europe and East Asia. Results indicated that Iran and Georgia had the highest number of female suicide cases in Western Asia (1). Some studies argue that self-immolation causes are different from other types of suicide. Several studies showed that middle-aged men with financial problems who had a history of psychiatric problems had the highest rate of suicide (8). Laloe identified three groups among those who had performed self-immolation, mentally ill individuals (West and Middle East) and those with personal (India, Sri Lanka, New Guinea, and Zimbabwe) and political reasons (India and South Korea) (7). The main cause of self-immolation in Hong Kong was financial problems, while in Taiwan the causes were separation, financial difficulties, and illness (9).

Differences in demographic patterns were also observed. Kato et al. in Japan (10), Lin et al. in Taiwan (9), and Chen et al. in England showed that self-immolation in men is more than women (11), while in Iran, it is more prevalent in women (3, 5, 12). Hashemiyan and Raghibi’s study showed that self-immolation is the second method of suicide in Sistan and Baluchistan, East of Iran (13). Regarding the frequency of self-immolation in Sistan and Baluchestan Province, a comprehensive study was performed on various aspects of self-immolation, results of which were noteworthy regarding cultural, social, religious texture of the region yet further studies and more comprehensive analysis are required in order to more precisely understand the reasons and find resolutions.

## 2. Objectives

Therefore, the aim of this study was to assess the rate and leading factors of self-immolation and gender, geographical distribution, and social norms of affected patients referred to Khatamolanbia Hospital of Zahedan from March 2010 to May 2012.

## 3. Materials and Methods

This descriptive and objective-based study was cross-sectional and retrospective with 750 burn patients; 315 of which had attempted self-immolation and most died. The data collection tool was a two partite questionnaire consisting of 17 questions (8 questions about personal details and 9 questions about self-immolation factors). The collected data were analyzed by the SPSS software.

## 4. Results

350 self-immolations resulting in death were reported in this study; they were 16-25 years old, 67.25% female, 63.55% had elementary school education, 74.20% married, 69.10% housewife, 61.05% resident of Saravan, 93.35% oil burns, 72.30% middle and low social class, 90.20% burn over 68%, 20% with psychiatric illness history and being treated with antidepressants, 73.25% verbal and physical violence before burning, and 100% of burnings were located inside a house and usually during the afternoon.

**Table 1.** Individual Specifications

	No. (%)
**Gender**	
Female	212 (67.25)
Male	103 (32.75)
Total	315 (100)
**Age**	
9-15	32 (10.14)
16-25	218 (69.33)
26-34	65 (20.53)
**Education**	
Illiterate	64 (20.15)
Elementary	201 (63.55)
Diploma and higher	50 (16.30)
Total	315 (100)
**Spouse or family violence**	
Physical or verbal violence	230 (73.25)
No violence	85 (26.75)
Total	315 (100)
**Occupation**	
Housewife	217 (69.10)
Employer	41 (13.30)
Free	57 (17.60)
Total	315 (100)

**Table 2.** Other Charecteristics of Clients^**a**^

	No. (%)
**Social Level (Income)**	
Weak	38 (12.30)
Middle	227 (72.30)
Good	50 (15.40)
Total	315 (100)
**Combustible material**	
Oil	294 (93.35)
Gas	20 (6.15)
Gasoline	1 (0.50)
Total	315 (100)
**Frequency of participants in terms of burn percentage**	
10-24	97 (2.30)
25-47	120 (3.05)
47-67	218 (4.45)
> 68	315 (90.20)
Total	750 (100)
**History of mental illness**	
Hospitalized	2 (0.35)
Drug consumption	63 (20.10)
Lack of both	250 (79.55)
Total	315 (100)
**Time**	
0-6	9 (3.15)
6-12	22 (6.87)
12-18	36 (11.23)
18-24	248 (78.75)
Total	315 (100)
**Cities of province**	
Nikshahr	16 (5.10)
Chahbahar	20 (6.25)
Khash	23 (7.30)
Zahedan	26 (8.22)
Iranshahr	39 (12.08)
Saravan	191 (61.05)
Total	315 (100)

^a^ Income; Over 5000 Rials Good, 5000 Rials Medium, Under 5000 Rials Low.

## 5. Discussion

Self-immolation and choosing this form of suicide requires analysis from several perspectives. Fire is used as a symbol or an available method. Sometimes self-immolation is committed to protest against political, social and economical issues. Those who burn themselves for this purpose sometimes lead other protestors to receive people or officials’ attention or to display their outrage. Self-inflicted burn, committed for any reasons, does irreparable damages to the person, their family and society.

There are differences between various societies in etiology, endangered groups and prediagnosis of self-immolated patients (3). In this study the patient’s mean age was 25-30 years, which is consistent with studies from other provinces (3, 5, 12, 14, 15). Studies show that the mean age of self-immolators in the world is one decade higher than that of Iran; this may be the result of different conditions dominat in Iran (11, 16, 17). The fact that most suicides are committed at a young age may be due to problems youth face and their wrongful understanding of ways to cope with stress and problems.

Also, a large portion of suicides by immolation were performed by women, similar to previous studies in which mean number of women was 54-81% (3, 5, 12, 14, 15, 18). In studies from China, Taiwan, and England, male were more than female (9-11, 16). Higher rate of self-immolation among women shows their insecure feelings attributed to the lack of training and supporting programs, poor economic condition, disputes and domestic violence. Therefore, one way to decrease this kind of suicide is to lend support to women in the family and society (3). Concerning education, most cases of self-immolation were committed by people with primary education, which is consistent with the study of Ahmadi et al.; set in Kermanshah (6), 84% of the subjects had elementary or lower education which was higher than education level of our subjects but consistent with the study of Rezaei et al. (18). Seventy-three percent of self-immolation cases occurred in families with physical or verbal aggression; other papers have not mentioned this fact. Similar to other studies, the frequency of housewives was high (3, 5, 14).

In this study, similar to previous reports, the most prevalent tool of self-immolation was the flame of inflammable liquids especially oil (3). The other important issue is the difference between the distribution of self-immolation cases between the towns of Sistan and Baluchestan; 61% of self-immolation cases are committed in the Saravan County. This requires further studies and investigation to find out why this town has such a high rate. In several studies, middle-aged men, who had performed self immolation, had financial problems (9). Laloe identified three groups: people who are mentally ill (western countries and Middle East) (7), people who have personal reasons (India, Sri Lanka, New Guiana and Zimbabwee) and people who commit self-immolation for political reasons (India and South Korea) (7). In Hong Kong, financial problems were the major reason for self-immolation. In Taiwan, separation, financial problems and illness were three reasons for self-immolation. In this study, 20% of people took psychiatric drugs and 74% of cases occurred in families with physical or verbal aggression.

Among the limitations of this study was the impossibility to study the reasons for committing self-immolation. Follow-up was impossible for the patients or their families due to their lack of interest, failure to find the whereabouts of patients or other reasons.

1. High rate of suicide attempt is one of the indicators of mental health and social disorder. Due to its high rate in this area, solutions for improvement of life quality and social norms should be reviewed and implemented.
2. Due to the significant geographical dispersion, prevention and research programs and guidelines should be more concentrated in some regions of the province including Saravan City.
3. Since familial problems may result in suicide, pre-marriage counseling centers should be established to improve correct and informed marriage culture and to encourage the couples to know each other before marriage.
4. Regarding suicide, religious beliefs should be strengthened and Islamic commands should be trained.
5. Anti-drug programs should be considered.
6. Mental health training should start at schools.
7. Legislation to protect women should be a priority for health programs.
8. All forms of mental disorders should be dealt with properly from the beginning.
9. Regarding high costs of treatment of these patients, much better results would be expected if only a small percentage of those costs were allocated to prevention.
10. Use of energy fuels like urban gas plumbing should be accelerated and oil should not be available (since the highest rate of self-immolation was with oil).
